# A curcumin-borneol prodrug delays rheumatoid arthritis progression by inhibiting the activation of the MAPK/AP-1-MMP9 inflammatory axis

**DOI:** 10.3389/fimmu.2026.1775735

**Published:** 2026-03-09

**Authors:** Tianyi Xu, Qilei Yang, Yue Peng, Congcong Xie, Huimin Yu

**Affiliations:** 1 Department of Traditional Chinese Medicine, The Second Affiliated Hospital of Harbin Medical University, Harbin, China; 2 Key Laboratory of Forest Plant Ecology, Ministry of Education, Northeast Forestry University, Harbin, China; 3 College of Chemistry, Chemical Engineering and Resource Utilization, Northeast Forestry University, Harbin, China; 4 Department of Traditional Chinese Medicine, The Second Affiliated Hospital of Zhejiang University School of Medicine, Hangzhou, China

**Keywords:** borneol, curcumin, MAPK/AP-1 signaling, matrix metalloproteinase-9 (MMP9), rheumatoid arthritis

## Abstract

Rheumatoid arthritis (RA) therapy demands agents that are both effective and safe, yet many natural products with promising bioactivity, such as curcumin, face major translational challenges due to poor solubility and bioavailability. To overcome these limitations, we designed and synthesized a series of dual-functional curcumin-borneol prodrugs via a succinate ester linkage, including the parent ester (CBS), its water-soluble sodium salt (CBS-Na), and a disubstituted analog (DCBS), to enhance delivery and synergize therapeutic action. These derivatives markedly improved cellular uptake and nuclear localization, potently scavenged reactive oxygen species, and effectively suppressed the MAPK/AP-1 signaling axis by inhibiting ERK and p38 phosphorylation and c-Fos expression, leading to downregulation of matrix metalloproteinase MMP9 and pro-inflammatory cytokines (TNF-α, IL-6, IL-1β). In a collagen-induced arthritis rat model, the derivatives demonstrated superior efficacy over native curcumin in alleviating joint inflammation and destruction, while exhibiting an excellent safety profile, thus representing a novel and promising therapeutic strategy for RA.

## Graphical Abstract

**Figure:**
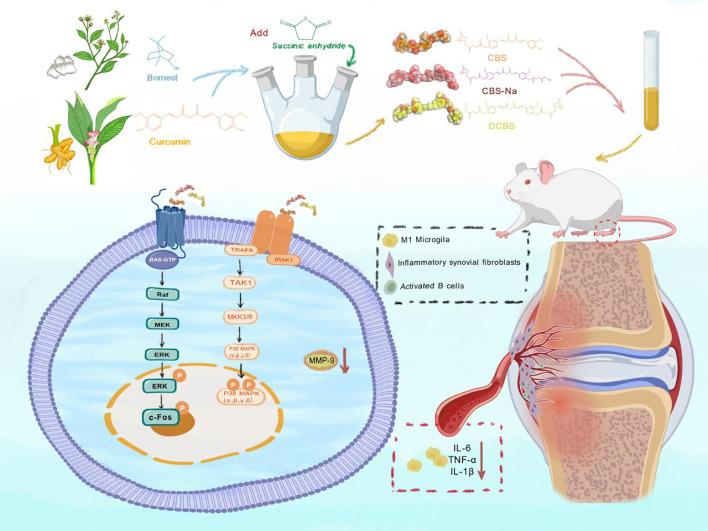
Illustrates the proposed mechanism through which curcumin-borneol ester derivatives ameliorate rheumatoid arthritis. The three derivatives were prepared through borneol esterification and subsequent salt modification. Owing to the superior membrane permeability and nuclear targeting capability conferred by the borneol moiety, these compounds are efficiently internalized into target cells. Intracellularly, they act synergistically to inhibit the phosphorylation of ERK and p38 within the MAPK signaling pathway, thereby downregulating the activity of the downstream transcription factor AP-1. This suppression subsequently leads to the reduced expression of matrix metalloproteinase-9 (MMP-9) and the pro-inflammatory cytokines IL-6, IL-1β, and TNF-α. Ultimately, this cascade of events achieves therapeutic efficacy by mitigating joint inflammation and bone destruction.

## 1 Introduction

Rheumatoid arthritis (RA) is a chronic, systemic autoimmune disorder characterized by persistent synovitis, synovial hyperplasia, and the progressive destruction of cartilage and bone, which frequently results in joint deformity and significant functional impairment (1–3). With a global prevalence of 0.5-1% and a higher prevalence in females, RA imposes a substantial burden on both patients’ quality of life and healthcare systems. Current therapeutic strategies, including non-steroidal anti-inflammatory drugs (NSAIDs), conventional disease-modifying antirheumatic drugs (DMARDs) such as methotrexate, and biologic agents (e.g., TNF-α inhibitors), are employed to provide symptomatic relief and modify the disease course (4, 5). However, the long-term application of these treatments is frequently hampered by significant limitations, including gastrointestinal, hepatic, and renal toxicities, an elevated risk of infections due to immunosuppression, and high costs (6, 7). Consequently, the development of safer, more effective, and affordable anti-RA agents remains an urgent and unmet medical need.

In response to these challenges, natural products, particularly active components derived from traditional Chinese medicine (TCM), have garnered significant attention as promising alternative or complementary therapies due to their multi-targeting capabilities and favorable.

safety profiles (8). Among them, curcumin, the principal polyphenolic compound from Curcuma longa, has demonstrated considerable potential for RA management. It exhibits potent anti-inflammatory, antioxidant, and anti-angiogenic properties by suppressing key signaling pathways such as NF-κB and MAPK, downregulating pro-inflammatory cytokines (e.g., TNF-α, IL-1β, IL-6), and ameliorating disease severity in various animal models of arthritis (9–12). Despite this promising pharmacological profile, the clinical translation of curcumin is severely hindered by its inherent physicochemical drawbacks, notably extremely poor aqueous solubility, low oral bioavailability, and rapid systemic metabolism and elimination (13). These limitations drastically reduce its effective concentration at the disease site, thereby undermining its therapeutic efficacy.

To directly address the critical issue of curcumin’s poor drug-like properties, a rational prodrug strategy centered on molecular hybridization with borneol was employed. Borneol, a well-known “guide drug” in TCM, is widely recognized for its ability to enhance drug penetration across biological barriers, such as the gastrointestinal mucosa and the blood-brain barrier, and to improve distribution to target tissues (14–18). It was hypothesized that covalently conjugating curcumin with borneol via a biodegradable succinate ester linker would create a dual-functional prodrug that synergistically combines the therapeutic action of curcumin with the enhanced delivery properties of borneol. This molecular design was anticipated to improve both lipophilicity for membrane permeation and hydrophilicity, via the ionizable carboxyl group of the succinate linker, for aqueous dispersibility (19–21). This approach yielded the lead compound, curcumin-borneol succinate ester (CBS). To further optimize the physicochemical properties, its sodium salt (CBS-Na) was developed to maximize water solubility, and a disubstituted analog (DCBS) was synthesized to augment borneol-mediated targeting and permeability.

Beyond the synthesis and characterization of these novel derivatives, the underlying mechanism of action was systematically investigated. Using network pharmacology, matrix metalloproteinase-9 (MMP9) was predicted to be a core target in RA pathology (22). MMP9 plays a pivotal role in cartilage breakdown by degrading type II collagen and other extracellular matrix components, and its expression is transcriptionally regulated by the MAPK/AP-1 axis (23–25). In the RA synovial microenvironment, MAPKs (including p38 and ERK) are hyperactivated, leading to the phosphorylation and nuclear translocation of AP-1 components (c-Fos/c-Jun), which in turn drive the expression of destructive enzymes like MMP9 (26, 27). Given that curcumin is known to modulate MAPK signaling (28), it was postulated that the novel curcumin-borneol ester derivatives-with their superior bioavailability and cellular uptake-would more effectively inhibit MAPK phosphorylation, suppress AP-1 activation, and consequently reduce MMP9 expression, thereby alleviating synovitis and halting joint erosion. To experimentally validate this mechanistic hypothesis concerning the MAPK/AP-1-MMP9 axis, a combination of *in vitro* assays and *in vivo* evaluation was required. For the latter, we employed the rat collagen-induced arthritis (CIA) model, a well-established preclinical tool that reliably recapitulates key pathophysiological features of human RA, including synovial inflammation, pannus formation, and progressive cartilage and bone erosion (29, 30).

Therefore, this study was designed to systematically evaluate the therapeutic potential and underlying mechanisms of these novel curcumin-borneol ester derivatives. To visually summarize our experimental workflow and proposed mechanism, we generated the graphical abstract using the Generic Diagramming Platform (GDP), a comprehensive database of biomedical graphics (31). Our comprehensive investigation encompassed: (a) the rational design, synthesis, and structural characterization of the derivatives; (b) *in vitro* assessment of their antioxidant capacity, cellular uptake, and modulation of the MAPK/AP-1 pathway; and (c) *in vivo* evaluation of their efficacy and safety in the CIA rat model. This multi-faceted approach aims not only to verify the potential of these derivatives as promising anti-RA drug candidates but also to validate the prodrug strategy itself.

## 2 Experimental section

### 2.1 Materials

Curcumin (used as the starting material), succinic anhydride, and sodium hydride were purchased from Shanghai Macklin Biochemical Co., Ltd. (-)-Borneol was obtained from Shanghai Aladdin Biochemical Technology Co., Ltd. All chemicals and solvents were of analytical grade and used without further purification unless otherwise specified.

### 2.2 Synthesis of curcumin-borneol ester derivatives

#### 2.2.1 Synthesis of curcumin-borneol succinate

The synthesis was initiated with the preparation of the bornyl succinate intermediate. Briefly, borneol was reacted with succinic anhydride in dichloromethane (DCM) at 40°C for 48 hours under catalysis by 4-dimethylaminopyridine (DMAP) and triethylamine. The reaction mixture was subsequently washed with water and brine to remove the catalysts, dried over anhydrous sodium sulfate, and concentrated under reduced pressure to afford a white solid. In the next step, curcumin was coupled with the synthesized bornyl succinate ester in DCM at 40°C for 48 hours using 1-ethyl-3-(3-dimethylaminopropyl) carbodiimide (EDC) and DMAP as coupling agents. Upon completion, the reaction mixture was washed, dried, and concentrated to obtain the crude product. Purification was achieved by silica gel column chromatography using a mixture of petroleum ether and ethyl acetate (2:1, v/v) as the eluent. The fractions containing the target compound were collected and concentrated via rotary evaporation to yield pure CBS.

#### 2.2.2 Preparation of curcumin-borneol succinate sodium salt

CBS was first reacted with an excess of succinic anhydride in the presence of a base to form a carboxylic acid intermediate. After aqueous work-up, the intermediate was isolated and then reacted with sodium hydride in tetrahydrofuran for 24 hours. The solvent was removed under reduced pressure, yielding the final sodium salt, CBS-Na, as a solid.

#### 2.2.3 Preparation of curcumin bis-borneol succinate

A similar synthetic procedure to that of CBS was utilized to prepare the disubstituted derivative, DCBS. The molar ratio of the reactants was adjusted to Curcumin: Bornyl Succinate =.

1:2. Under identical reaction and purification conditions, DCBS was obtained.

### 2.3 Structural characterization

The structures of the synthesized derivatives (CBS, CBS-Na, DCBS) were characterized by Fourier Transform Infrared (FT-IR) spectroscopy, Ultraviolet-Visible (UV-Vis) spectrophotometry, and Nuclear Magnetic Resonance (NMR) spectroscopy. FT-IR spectra were recorded on a Thermo Scientific iS5 spectrometer. UV-Vis analysis was conducted using a Shimadzu UV2600 spectrophotometer. 1H NMR spectra were acquired on a Bruker BioSpin GmbH spectrometer (Topspin version 3.2) using deuterated dimethyl sulfoxide (DMSO-*d*_6_, Cambridge Isotope Laboratories) as the solvent.

### 2.4 Cell culture

RAW 264.7 mouse macrophages and Caco-2 human colon adenocarcinoma cells were obtained from Procell (Wuhan, China). RAW 264.7 cells were cultured in Dulbecco’s Modified Eagle Medium (DMEM, Gibco, USA) supplemented with 10% fetal bovine serum (FBS). Caco-2 cells were maintained in DMEM containing 20% FBS. All cell lines were cultured at 37°C in a humidified atmosphere containing 5% CO_2_.

### 2.5 *In vitro* assays

#### 2.5.1 Live/dead staining

Caco-2 cells were seeded in 12-well plates at a density of 60,000 cells per well and incubated overnight. The cells were then treated with 4.0 mg/mL of curcumin (CUR), CBS, CBS-Na, or DCBS for 12 hours. Following treatment, cell viability was assessed using a Live/Dead cell staining kit (Biosharp, China) according to the manufacturer’s protocol. Specifically, cells were stained with calcein AM (green fluorescence) to label live cells and propidium iodide (red fluorescence) to label dead cells. The stained cells were immediately visualized using a high-content cell imaging system (BioTek Instruments, Inc., USA).

#### 2.5.2 Cellular uptake assay

RAW 264.7 cells were plated in 24-well plates at a density of 30,000 cells per well and allowed to adhere overnight. After treatment with the test compounds, the cells were fixed and stained with 4’,6-diamidino-2-phenylindole (DAPI, C1002, Beyotime, China) for 10 minutes to visualize nuclei. Cellular uptake was observed under a high-content cell imaging system (BioTek Instruments, Inc., USA), and the mean fluorescence intensity was quantified using ImageJ software (National Institutes of Health, USA).

#### 2.5.3 Intracellular ROS detection

RAW 264.7 cells were seeded in a 96-well plate at a density of 10,000 cells per well and cultured overnight. After the designated treatments, the cells were incubated with 10 μM 2’,7’-dichlorodihydrofluorescein diacetate (DCFH-DA, S0033, Beyotime, China) for 20 minutes at 37°C. Intracellular reactive oxygen species (ROS) levels, indicated by DCF fluorescence, were measured using a high-content cell imaging system. The average fluorescence intensity per well was calculated and analyzed using ImageJ software.

#### 2.5.4 Western blot analysis

RAW 264.7 cells were cultured in 6-well plates at a density of 400,000 cells per well overnight. Following treatment, total protein was extracted using RIPA lysis buffer (PC102, Epizyme, China), and the protein concentration was determined with a bicinchoninic acid (BCA) protein assay kit (ZJ101, Epizyme, China). Equal amounts of protein (20-30 μg per lane) were separated by SDS-polyacrylamide gel electrophoresis (SDS-PAGE, G2075, Servicebio, China) and transferred onto polyvinylidene fluoride (PVDF) membranes (G6046-0.45, Servicebio, China). The membranes were blocked with 5% (w/v) skim milk for 1 hour at room temperature and subsequently incubated overnight at 4°C with the following primary antibodies from Servicebio (China): GAPDH (1:1000), ERK (1:600), phospho-ERK (p-ERK, 1:600), p38 (1:1000), phospho-p38 (p-p38, 1:1000), and c-Fos (1:1000). After washing, the membranes were incubated with horseradish peroxidase (HRP)-conjugated secondary antibodies (1:10,000, Servicebio, China) for 1 hour at room temperature. Protein bands were visualized using an Ultra-Sensitive Enhanced Chemiluminescence (ECL) reagent kit (G2020, Servicebio, China) and imaged with a Tanon Chemi Dog Ultra system (TANON, China). Band intensities were quantified using ImageJ software.

### 2.6 Network pharmacology and molecular docking

#### 2.6.1 Active ingredient screening and target prediction

The active ingredients of curcumin and borneol were retrieved from TCMSP using OB ≥ 20% and DL ≥ 0.12 as criteria. Their SMILES notations were acquired from PubChem, and 10 components (7 from curcumin, 3 from borneol) were confirmed via SwissADME. Targets were predicted using SwissTargetPrediction, yielding 331 potential targets.

#### 2.6.2 Disease target collection and intersection analysis

RA-related targets were collected from GeneCards and OMIM databases, resulting in 1,674 targets after deduplication. The intersection with drug targets yielded 139 common targets via Venny 2.1.0.

#### 2.6.3 PPI network construction and hub target identification

The 139 common targets were imported into STRING (confidence > 0.4) to construct a PPI network, visualized and analyzed with Cytoscape. Topological analysis using the Centiscape plugin identified 29 hub targets.

#### 2.6.4 Molecular docking

3D structures of 10 key targets (e.g., MMP9, MAPK1) were obtained from the PDB. Structures of CBS, CBS-Na, and DCBS were built with Chem3D. Molecular docking simulations were carried out using AutoDock Vina. The binding affinity between each derivative and the target protein was evaluated and is presented as the predicted binding free energy (ΔG, kcal/mol), with more negative values indicating stronger binding.

### 2.7 *In vivo* efficacy and safety evaluation

#### 2.7.1 Animals and CIA model establishment

Based on its translational relevance to human RA pathology as described above, the CIA model was employed. Healthy Specific Pathogen-Free (SPF) grade Sprague-Dawley (SD) rats (7–8 weeks old, 200–300 g) were sourced from the Experimental Animal Center of the Second Affiliated Hospital of Harbin Medical University. All animal experiments were approved by the Institutional Animal Care and Use Committee (IACUC) (Approval No. YJSDW2024-154). The Collagen-Induced Arthritis (CIA) model was established by intradermally injecting 0. 1 mL of a 1:1 emulsion of bovine type II collagen (S31372, Orileaf, China) and Complete Freund’s Adjuvant (Beyotime, China) at the base of the tail on Day 0. A booster immunization was administered on Day 14 with 0.1 mL of a 1:1 emulsion of collagen and Incomplete Freund’s Adjuvant (Beyotime, China). CIA rats were randomly assigned to one of eleven experimental groups. Starting on day 29, the groups received daily oral administrations of either saline, methotrexate (MTX), total glucosides of paeony (TGP), curcumin (CUR), CBS, CBS-Na, or DCBS. Hind paw thickness was measured with a caliper every three days from day 28. Arthritis scores and body weights were assessed and documented on day 43. On the terminal day, blood serum, liver, spleen, kidney, and ankle joint tissues were collected for subsequent analysis.

#### 2.7.2 Animal grouping and drug administration

Sixty rats were randomly divided into the following 11 groups (n=5 per group).

Group 1: Ctrl (Blank control): Vehicle.

Group 2: Model (CIA model): Vehicle.

Group 3: MTX: Methotrexate, 0.6 mg/kg.

Group 4: TGP: Total glucosides of paeony, 78 mg/kg.

Group 5: CUR: Curcumin, 500 mg/kg.

Group 6: CBS-L: Curcumin-borneol succinate low dose, 500 mg/kg.

Group 7: CBS-H: Curcumin-borneol succinate high dose, 1000 mg/kg.

Group 8: CBS-Na-L: CBS sodium salt low dose, 500 mg/kg.

Group 9: CBS-Na-H: CBS sodium salt high dose, 1000 mg/kg.

Group 10: DCBS-L: Curcumin bis-borneol succinate low dose, 500 mg/kg.

Group 11: DCBS-H: Curcumin bis-borneol succinate high dose, 1000 mg/kg.

#### 2.7.3 Histopathological and immunohistochemical analysis

Ankle joint tissues were fixed in 4% paraformaldehyde for 48 hours, decalcified in 10% ethylenediaminetetraacetic acid (EDTA) for one month, processed, embedded in paraffin, and sectioned into 3 μm thick slices. The sections were stained with Hematoxylin and Eosin (H&E) and Safranin O/Fast Green for histopathological evaluation. For IHC, paraffin-embedded sections were dewaxed, subjected to antigen retrieval, blocked with serum, and incubated overnight at 4°C with primary antibodies against p-ERK1/2, c-Fos, c-Jun, and MMP9. This was followed by incubation with appropriate horseradish peroxidase (HRP)-conjugated secondary antibodies. Signal development was performed using a diaminobenzidine (DAB) substrate, and images were captured and analyzed using ImageJ software. Liver and kidney tissues were similarly processed and stained with H&E to evaluate pathological changes.

#### 2.7.4 Organ index and serum cytokine analysis

The liver and spleen indices were calculated according to the following formulae:

Liver Index (%) = [Liver Wet Weight (g)/Final Body Weight (g)] × 100%.

Spleen Index (%) = [Spleen Wet Weight (g)/Final Body Weight (g)] × 100%.

Serum levels of tumor necrosis factor-alpha (TNF-α), interleukin-6 (IL-6), and interleukin-1 beta (IL-1β) were quantified using specific enzyme-linked immunosorbent assay (ELISA) kits (LunChangShuo Biotech, China) in strict accordance with the manufacturers’ protocols.

### 2.8 Statistical analysis

All data are expressed as the mean ± standard deviation (SD). Statistical comparisons between two groups were performed using an unpaired, two-tailed Student’s t-test. For comparisons among multiple groups, one-way analysis of variance (ANOVA) was employed, followed by an appropriate *post-hoc* test. A p-value below 0.05 was considered to indicate statistical significance (*p < 0.05).

## 3 Results and discussion

### 3.1 Synthesis and structural confirmation of curcumin-borneol ester derivatives

#### 3.1.1 Chemical synthesis

Three novel curcumin-borneol ester derivatives were synthesized based on a rational prodrug strategy. The synthesis was initiated with the preparation of a key intermediate, borneol succinate monoester, via a DMAP/triethylamine-catalyzed ring-opening reaction of borneol with succinic anhydride under anhydrous conditions. This intermediate was subsequently coupled with curcumin using EDC and DMAP to afford curcumin-borneol succinate ester (CBS). To enhance aqueous solubility, CBS was converted into its sodium salt (CBS-Na) through a reaction with sodium hydride. Additionally, a disubstituted analog, curcumin bis-borneol succinate ester (DCBS), was synthesized by reacting curcumin with two equivalents of the borneol succinate intermediate under similar conditions. All target compounds were purified and their structures were unequivocally confirmed prior to biological evaluation.

#### 3.1.2 Structural characterization

The molecular structures of CBS, CBS-Na, and DCBS were systematically confirmed by FT-IR, UV-Vis, and ¹H NMR spectroscopy.

**Figure 1:**
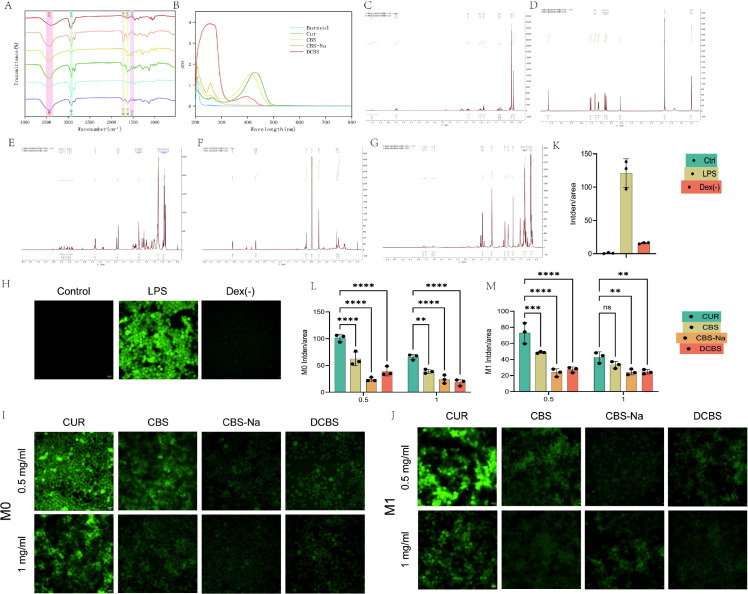
Synthesis and characterization of curcumin-borneol ester derivatives. **(A)** FT-IR spectra. **(B)** UV-Vis absorption spectra. **(C-G)** ¹H NMR spectra. **(C)** Borneol **(D)** Curcumin **(E)** CBS **(F)** CBS-Na **(G)** DCBS **(H-J)** Representative images of intracellular ROS detection using the DCFH-DA fluorescent probe (scale bar = 15 μm). **(K-M)** Quantitative analysis of ROS fluorescence intensity. The M1 group represents treatments under LPS stimulation. Data are presented as mean ± SD (n=3). *p < 0.05, **p < 0.01, ***p < 0.001 vs. LPS group; ns, not significant.

FT-IR analysis (**Figure 1A**) confirmed successful ester bond formation. The characteristic strong anhydride C=O stretch of succinic anhydride disappeared, while a new, strong ester C=O vibration emerged in the borneol succinate intermediate and the final derivatives. Concurrently, the broad O-H stretching vibration of curcumin’s phenolic groups (around 3,407 cm^-^¹) was notably diminished. Key characteristic absorptions of the curcumin core, such as the enol C=O stretch and aromatic C=C vibrations, were preserved in all derivatives. Furthermore, the intense aliphatic C-H stretching vibrations from the borneol moiety were clearly present in CBS, CBS-Na, and DCBS.

UV-Vis spectroscopy (**Figure 1B**) revealed distinct electronic transitions consistent with structural modifications. Compared to native curcumin (λmax = 426 nm), CBS exhibited a blue-shifted absorption maximum (λmax = 412 nm), indicating a perturbation of the π-conjugation by the borneol-succinyl linker. CBS-Na displayed new absorption peaks at 208 nm and 257 nm, attributed to carboxylate ionization and possible tautomeric shifts, accompanied by the disappearance of the characteristic 426 nm curcumin band. DCBS showed a broad absorption centered at 252 nm with a weaker band at 398 nm, characteristic of a symmetric bis-esterified system with shortened effective conjugation.

¹H NMR analysis (**Figures 1C–G**) provided conclusive evidence for structural integrity. In the spectrum of CBS, the characteristic phenolic proton signal of curcumin (δH ~9.66) was absent, confirming esterification. New signals corresponding to the borneol moiety and the succinyl linker (methylene protons at δH ~2.50) were present alongside the preserved aromatic and olefinic proton signals of the curcumin core. For CBS-Na, the upfield shift of the succinyl methylene protons (δH ~2.95) and the absence of carboxylic acid proton signals supported sodium salt formation. The DCBS spectra showed intensified signals in the high-field region (δH 0.8-2.0), consistent with the presence of two borneol units, corroborating the diester structure.

### 3.2 *In vitro* immunomodulatory effects of curcumin-borneol ester derivatives

Following the structural confirmation, the intracellular antioxidant activity of the derivatives was evaluated in LPS-stimulated RAW264.7 macrophages by measuring ROS levels with the DCFH-DA probe.

The DCF fluorescence assay results (**Figures 1H–M**) demonstrated that structural modification significantly enhanced the ROS-scavenging capacity of curcumin in a dose and structure dependent manner.

All three derivatives (CBS, CBS-Na, and DCBS) showed superior antioxidant activity compared to native curcumin. At low concentrations, CBS-Na was particularly effective, reducing intracellular ROS fluorescence to only 24.8% of the level observed with curcumin, a performance approaching that of dexamethasone.

The dose-response profiles differed between derivatives. CBS-Na exhibited a marked low-dose saturation effect; doubling its concentration resulted in minimal further reduction in fluorescence. In contrast, DCBS showed a clear dose-dependent enhancement, achieving up to an 84.1% reduction relative to the positive control at higher doses.

Under LPS-induced inflammatory (M1) conditions, all derivatives maintained significant activity. CBS-Na displayed excellent stability across models, showing similar efficacy in M0 and M1 macrophages, and further reduced ROS at high doses in the M1 state. DCBS performed strongly at low concentrations under M1 conditions, although its efficacy plateaued at higher doses.

### 3.3 Analysis of cellular uptake efficiency

Following the assessment of antioxidant activity, cellular uptake of the derivatives was evaluated in RAW264.7 macrophages over 2 and 4 hours using the intrinsic fluorescence of the curcumin scaffold.

**Figure 2:**
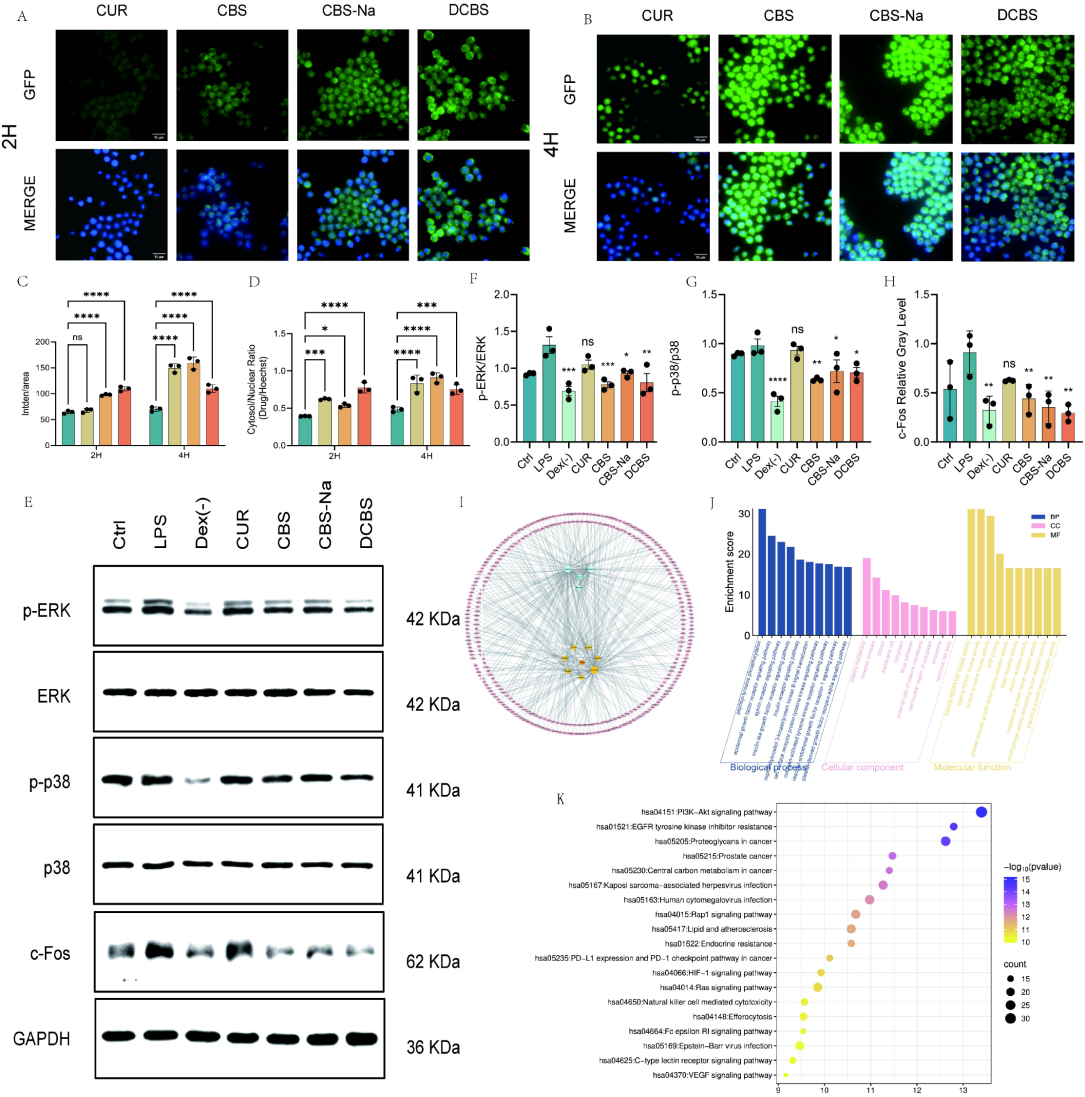
Cellular uptake and *in vitro* immunomodulatory properties of curcumin-borneol ester derivatives. **(A, B)** Representative immunofluorescence images (green: derivative; blue: Hoechst nuclear stain) of RAW264.7 cells after 2 h and 4 h incubation (scale bar = 15 μm). **(C)** Quantitative analysis of cellular fluorescence intensity at 2 h and 4 (h) **(D)** Analysis of the nuclear-to-cytoplasmic fluorescence ratio at 2 h and 4 (h) **(E-H)** Western blot analysis of ERK, p-ERK, p38, p-p38, and c-Fos protein levels and their quantitative analysis. **(I)** “Traditional Chinese Medicine-Component-Target-Disease” interaction network. **(J)** Gene Ontology (GO) enrichment analysis of potential targets. **(K)** Kyoto Encyclopedia of Genes and Genomes (KEGG) pathway enrichment analysis. Data are presented as mean ± SD (n=3). *p < 0.05, **p < 0.01, ***p < 0.001 vs. LPS group; ns, not significant.

All derivatives exhibited significantly higher cellular fluorescence intensity than native curcumin at both 2 and 4 hours (**Figures 2A–D**). The disubstituted analog DCBS showed the most rapid uptake, with a fluorescence intensity 1.65-fold higher than curcumin at the 2-hour time point. In contrast, CBS-Na demonstrated the highest cellular accumulation by 4 hours.

The nuclear-to-cytoplasmic fluorescence ratio was markedly increased for all derivatives compared to curcumin. DCBS achieved a ratio more than double that of the parent compound within 2 hours. The uptake kinetics revealed distinct patterns: DCBS reached maximal internalization and nuclear localization within 2 hours, indicating rapid saturation. Both CBS and CBS-Na showed progressive, time-dependent cellular accumulation over 4 hours.

### 3.4 Immunomodulatory mechanism of curcumin-borneol ester derivatives

To assess whether the enhanced cellular uptake translated into functional modulation of inflammatory signaling, the effects of the derivatives on the MAPK/AP−1 pathway were evaluated in LPS−stimulated RAW264.7 macrophages.

Western blot analysis confirmed that LPS stimulation significantly increased the phosphorylation of ERK and p38 and upregulated the expression of the AP−1 component c−Fos, indicating activation of the MAPK/AP−1 axis (**Figure 2E**). Total ERK and p38 protein levels remained unchanged across groups.

All three derivatives significantly inhibited ERK phosphorylation (**Figure 2F**). The disubstituted derivative DCBS showed the strongest suppression, reducing the p−ERK/ERK ratio close to baseline levels. CBS and CBS−Na also exhibited clear inhibitory effects.

In the p38 pathway, all curcumin-borneol ester derivatives consistently and significantly suppressed phosphorylation, whereas native curcumin had only a marginal effect (**Figure 2G**). CBS−Na and DCBS displayed the most potent inhibition of p−p38.

At the downstream transcriptional level, each derivative effectively reversed LPS−induced c−Fos expression (**Figure 2H**). DCBS produced the most pronounced reduction, lowering c−Fos below the level observed in the dexamethasone control group. CBS−Na and CBS also significantly suppressed c−Fos expression.

### 3.5 Mechanism research based on network pharmacology

The constructed compound - target - disease network consists of 343 nodes and 422 edges (**Figure 2I**), reflecting the multi-target characteristics of these derivatives. Enrichment analysis identified 837 significant GO terms, mainly related to inflammatory response and kinase activity, as well as 150 significantly enriched KEGG pathways (**Figures 2J, K**). Notably, the Ras signaling pathway (an upstream regulator of the MAPK cascade reaction) and multiple pathways related to MAPK were significantly enriched, supporting the view that the regulatory mechanism centered on MAPK plays a core role.

**Figure 3:**
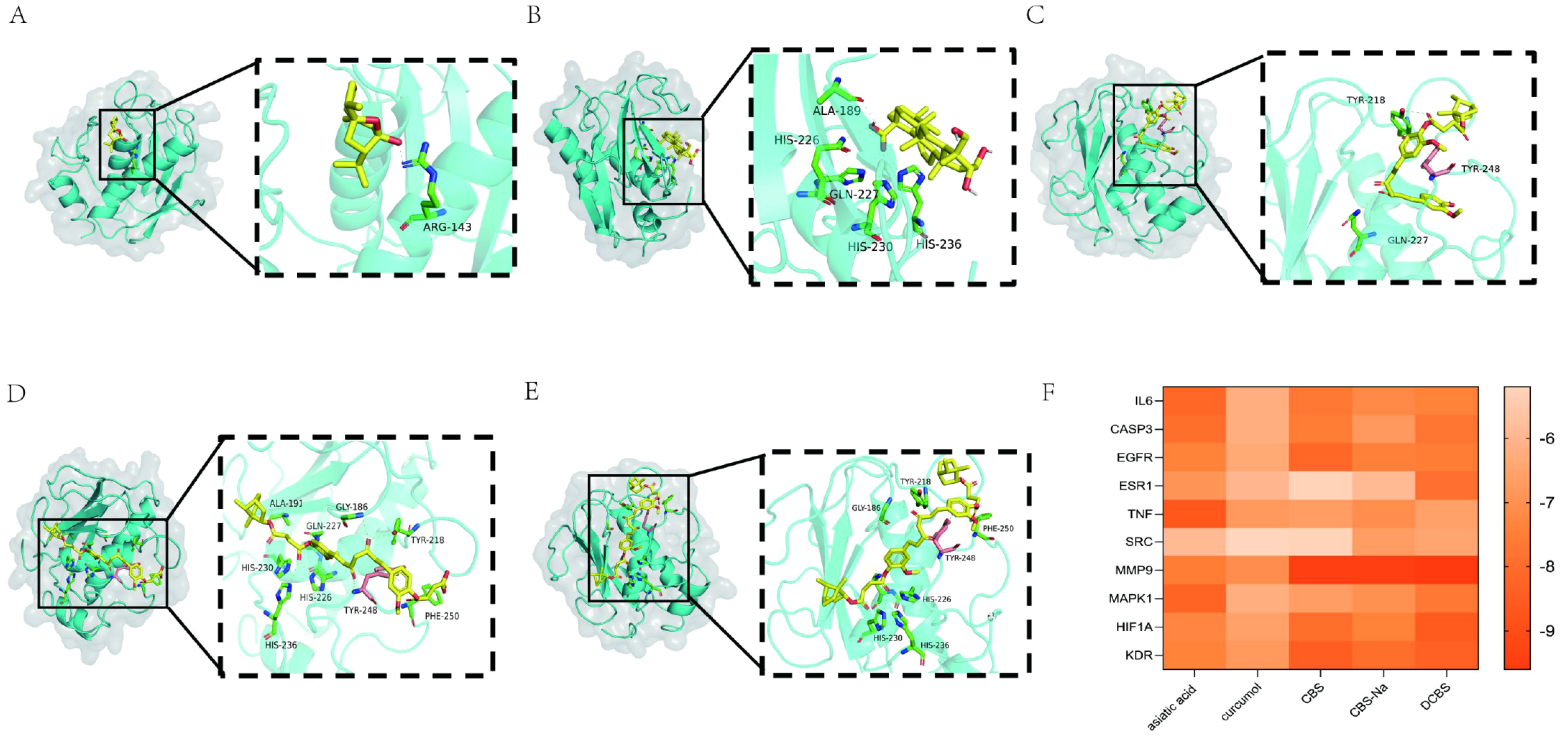
Molecular docking analysis. **(A-E)** Visualization of the molecular docking of drugs with MMP9 protein molecules: **(A)** Curcumol **(B)** Asiatic acid **(C)** CBS **(D)** CBS-Na **(E)** DCBS. **(F)** Heatmap of binding energies (kcal/mol) between the active compounds and key target proteins.

Molecular docking showed that these derivatives have strong binding affinity with key targets (**Figures 3A–E**). MMP9 showed the highest predicted binding affinity (lowest binding energy) among the targets, with all tested compounds exhibiting binding energies below -7.0 kcal/mol; DCBS had the lowest value (-9.6 kcal/mol) (**Figure 3F**).

### 3.6 *In vivo* therapeutic efficacy of curcumin-borneol ester derivatives

**Figure 4:**
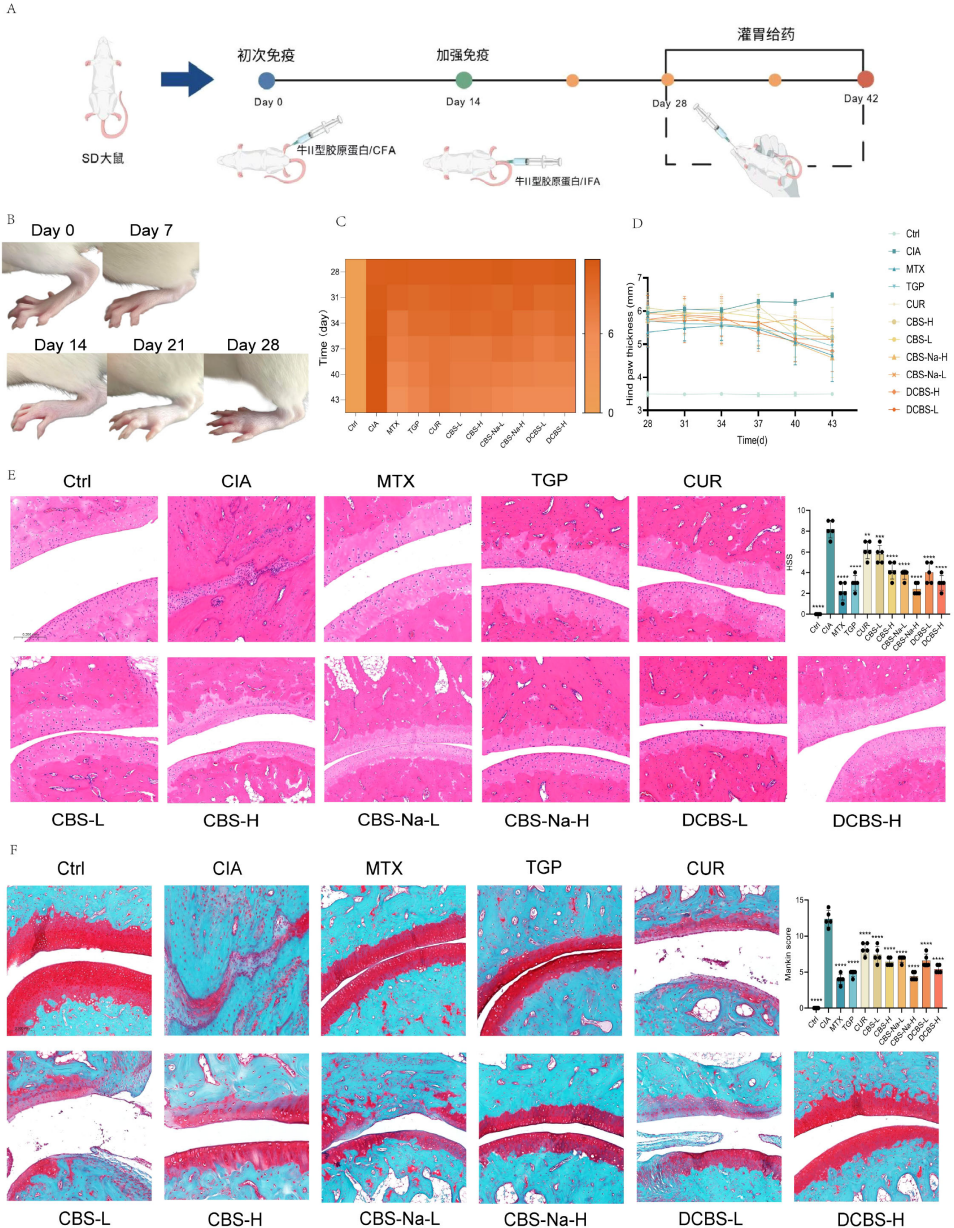
Therapeutic effects of Curcumin-Borneol Ester Derivatives in CIA rats. **(A)** Schematic diagram of the experimental protocol. **(B)** Macroscopic appearance of hind paws from different groups. **(C)** Heatmap analysis of arthritis scores over time. **(D)** Quantitative analysis of hind paw thickness. **(E)** Representative H&E-stained sections of ankle joints and quantitative analysis of the histopathological synovitis score (HSS). **(F)** Representative Safranin O/Fast Green-stained sections of ankle joints and quantitative Mankin scores for cartilage damage. Data are presented as mean ± SD (n=5). *p < 0.05, **p < 0.01, ***p < 0.001 vs. CIA model group; ns, not significant.

Building upon the mechanistic insights from network pharmacology, the anti-arthritic efficacy of the derivatives was further validated in a rat CIA model. Successful induction of arthritis was confirmed by the development of characteristic joint redness, swelling, and impaired mobility (**Figure 4B**).

All curcumin-borneol ester derivatives induced a marked, dose-dependent reduction in arthritis scores compared to the model group, with high-dose treatments showing efficacy comparable to methotrexate (MTX) (**Figure 4C**). Native curcumin showed only limited improvement.

Consistent with the clinical scoring, derivative treatment significantly attenuated joint edema (**Figure 4D**). Paw thickness measurements revealed a gradient of improvement: low-dose groups (CBS-L, CBS-Na-L, DCBS-L) showed clear therapeutic effects, while high-dose groups (CBS-H, CBS-Na-H, DCBS-H) produced the most pronounced anti-edema response. The CBS-Na-H and DCBS-H groups, in particular, restored paw thickness to near-normal levels, matching the efficacy of MTX and significantly outperforming the native curcumin group.

Histopathological evaluation via H&E staining corroborated these findings (**Figure 4E**). While the model group exhibited severe synovial hyperplasia, inflammatory infiltration, and cartilage erosion, all derivative-treated groups showed dose-dependent protection. Low-dose treatments effectively controlled synovial hyperplasia and inflammation, and high-dose groups (CBS-H, CBS-Na-H, DCBS-H) maintained near-normal joint architecture, with protective effects comparable to MTX and superior to native curcumin.

### 3.7 Cartilage-protective effects of curcumin-borneol ester derivatives

Safranin O/Fast Green staining was employed to assess the impact of the derivatives on cartilage integrity (**Figure 4F**). The CIA model group exhibited severe proteoglycan loss and cartilage structure disruption. In contrast, treatment with all three curcumin−borneol derivatives resulted in a dose−dependent preservation of cartilage architecture and proteoglycan content. High−dose treatments (CBS−H, CBS−Na−H, DCBS−H) showed the most pronounced effect, with cartilage morphology and staining intensity restored to levels comparable to the positive control (MTX). All derivative−treated groups demonstrated superior chondroprotection relative to the native curcumin group. Histopathological scoring confirmed the significant improvement in cartilage integrity afforded by the derivatives.

### 3.8 Tissue-level validation of MAPK/AP-1-MMP9 axis inhibition

**Figure 5:**
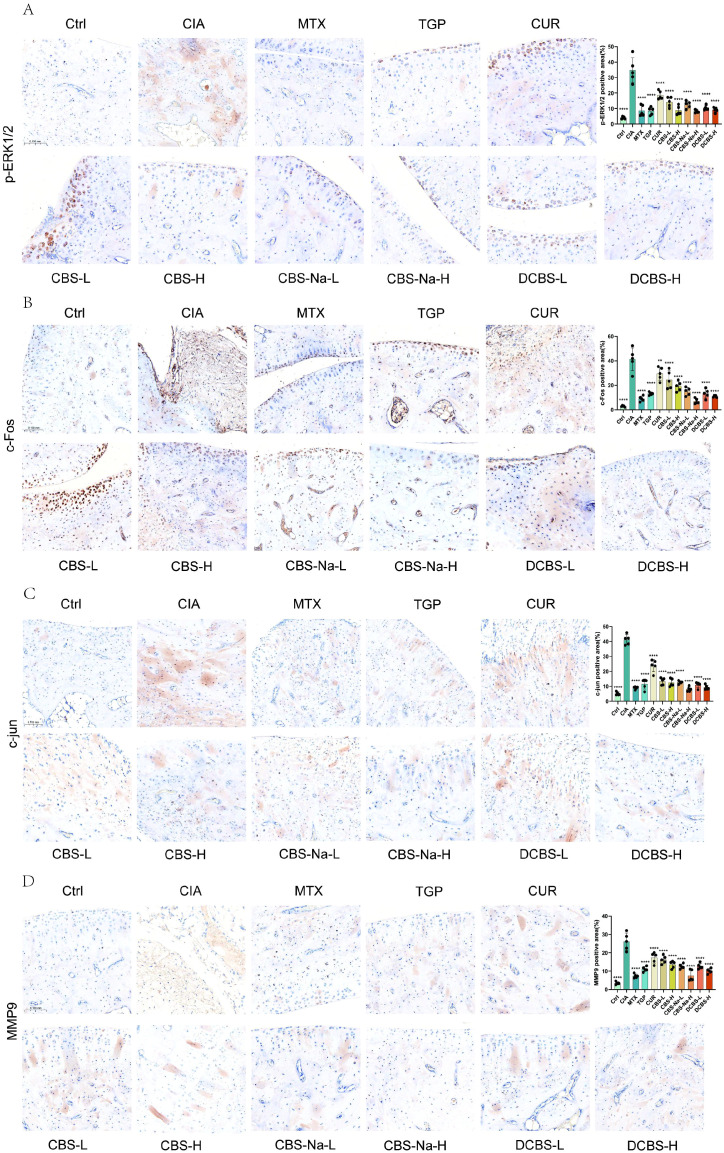
Inhibition of the MAPK/AP-1-MMP9 axis in joint tissues. **(A)** Representative IHC images of p-ERK1/2 in ankle synovium and quantitative analysis of p-ERK1/2-positive cells. **(B)** IHC for c-Fos and quantitative analysis. **(C)** IHC for c-Jun and quantitative analysis. **(D)** IHC for MMP9 and quantitative analysis. Data are presented as mean ± SD (n=5). *p < 0.05, **p < 0.01, ***p < 0.001 vs. CIA model group; ns, not significant.

To further delineate the molecular alterations underlying the observed therapeutic effects, immunohistochemical analysis was performed on ankle joint sections from CIA rats (**Figure 5**).

Strong expression of phosphorylated ERK1/2 (p−ERK1/2), c−Fos, c−Jun, and MMP9 was observed in the synovial and peri−cartilaginous regions of the model group (**Figures 5A–D**). Treatment with the curcumin-borneol ester derivatives resulted in a dose-dependent reduction in the expression of all four markers.

High−dose derivative groups (CBS−H, CBS−Na−H, DCBS−H) exhibited the most potent suppression. Staining intensity and distribution of p−ERK1/2 were markedly diminished, approaching levels seen in the MTX−treated group. Nuclear accumulation of the AP−1 components c−Fos and c−Jun was significantly reduced, with CBS−Na−H showing particularly effective control. MMP9 expression, notably strong at the synovial−cartilage junction in the model group, was profoundly inhibited, especially in the DCBS−H group. Low−dose treatments (CBS−L, CBS−Na−L, DCBS−L) also produced clear, albeit weaker, reductions in marker expression compared to the model group.

### 3.9 Systemic anti-inflammatory effects assessed by ELISA

Following the tissue-level analysis of signaling pathways, the systemic anti-inflammatory effects of the derivatives were assessed by measuring serum cytokine levels in CIA rats.

**Figure 6:**
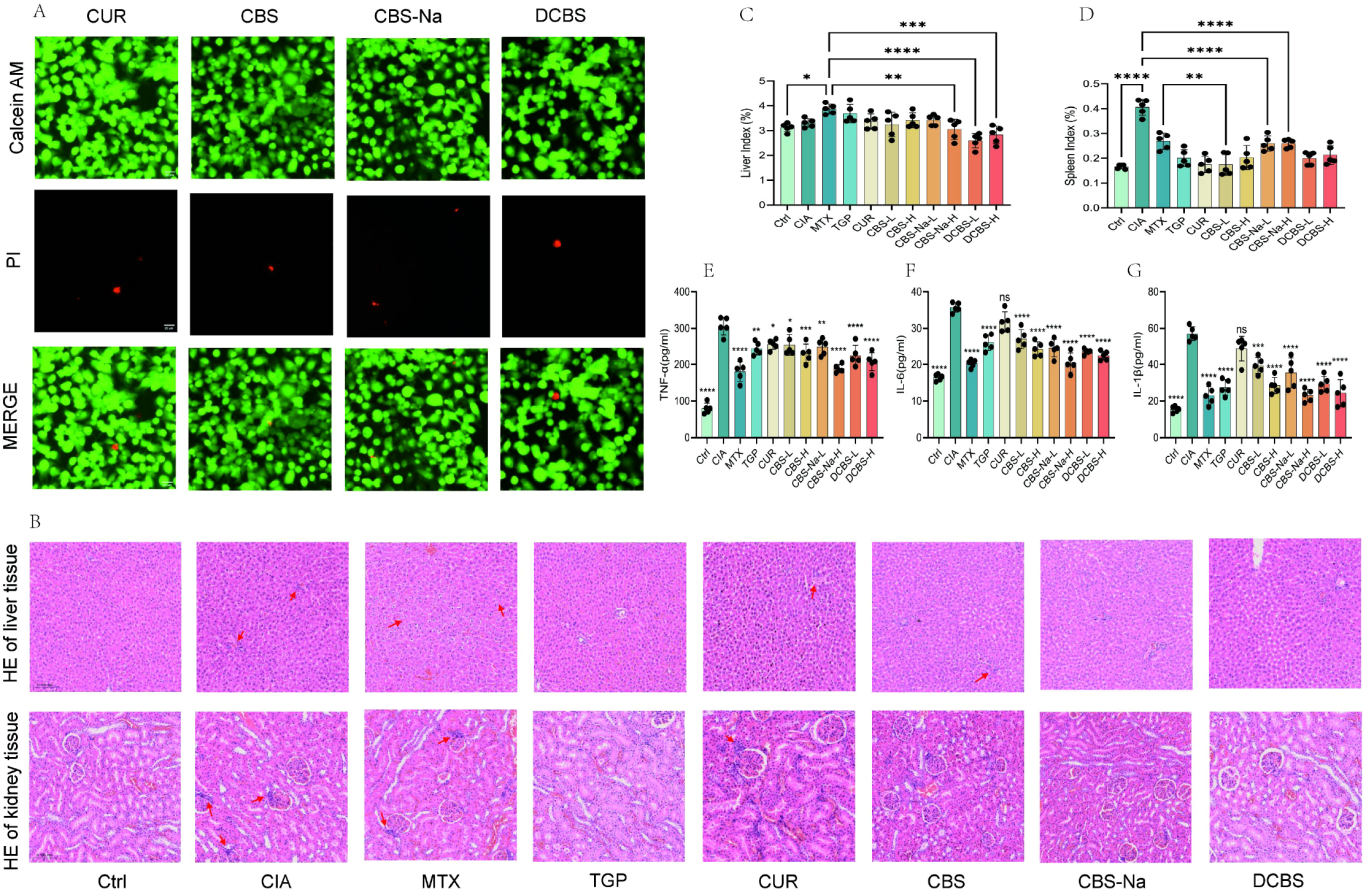
Safety evaluation and serum cytokine analysis. **(A)** Live/Dead cell staining of Caco-2 cells after treatment (scale bar = 20 μm). **(B)** Representative H&E-stained sections of liver and kidney tissues. **(C)** Quantitative analysis of the liver index. **(D)** Quantitative analysis of the spleen index. **(E-G)** ELISA analysis of serum TNF-α, IL-6, and IL-1β levels. Data are presented as mean ± SD (n=5). *p < 0.05, **p < 0.01, ***p < 0.001 vs. CIA model group; ns, not significant.

Serum levels of TNF−α, IL−6, and IL−1β were significantly elevated in the CIA model group, confirming a systemic inflammatory state. Treatment with the curcumin−borneol ester derivatives resulted in a distinct suppression profile for each cytokine (**Figures 6E–G**).

TNF−α levels were most potently reduced by MTX. Among the derivatives, the high−dose DCBS group (DCBS−H) showed significant inhibition, exceeding the effect of native curcumin. The high−dose CBS (CBS−H) and CBS−Na (CBS−Na−H) groups also produced clear reductions (**Figure 6E**).

For IL−6, all derivatives demonstrated substantial efficacy. The CBS−Na−H group exhibited inhibition nearly matching that of MTX and significantly surpassing curcumin. CBS−H and DCBS−H also showed strong suppressive effects (**Figure 6F**).

IL−1β levels were effectively lowered by the derivatives. Both the DCBS−H and CBS−Na−H groups exhibited exceptional inhibition, comparable to or even exceeding the effect of the MTX group. The CBS−H group also showed a significant reduction (**Figure 6G**).

### 3.10 Biosafety evaluation of curcumin-borneol ester derivatives

Following the assessment of therapeutic efficacy, the biosafety of the derivatives was evaluated at cellular and systemic levels.

#### 3.10.1 Cytotoxicity assessment

Live/dead staining in Caco−2 cells, a model of intestinal barrier function relevant to oral administration, revealed no significant cytotoxicity for curcumin or any of the derivatives at 10 μM. The majority of cells remained viable under these conditions (**Figure 6A**).

#### 3.10.2 *In vivo* safety profile

Histopathological analysis of liver and kidney tissues from CIA rats showed normal morphology in the blank control group, whereas the model group exhibited hepatic inflammatory infiltration and renal interstitial lesions (**Figure 6B**). The MTX group displayed hepatic steatosis and renal inflammation. In contrast, curcumin and all derivative-treated groups showed markedly milder pathological alterations, with tissue architecture in the CBS−Na and DCBS groups appearing nearly normal.

Organ index analysis further supported these observations (**Figures 6C, D**). Elevated liver and spleen indices in the model group, indicative of systemic inflammation, were effectively attenuated by treatment with CBS, CBS−Na, and DCBS. The spleen index recovered to near−normal levels in all derivative−treated groups, with DCBS showing the most pronounced effect. Liver indices in the CBS−Na and DCBS groups remained comparable to the normal range.

## 4 Discussion

This study demonstrates that the rational prodrug design and synthesis of curcumin−borneol ester derivatives (CBS, CBS−Na, and DCBS) significantly enhance the therapeutic potential against rheumatoid arthritis (RA). The structural modifications, confirmed by comprehensive spectroscopic characterization, successfully improved the physicochemical and pharmacokinetic properties of curcumin, particularly its cellular uptake and distribution. This enhancement translated into superior biological activity, as evidenced by potent antioxidant effects, efficient suppression of the MAPK/AP−1 signaling axis (specifically ERK and p38 phosphorylation and downstream c−Fos expression), and strong binding affinity to the key effector MMP9, as predicted by network pharmacology and validated by molecular docking.

In the CIA rat model, the derivatives exhibited dose−dependent efficacy in alleviating clinical arthritis scores, joint swelling, and histopathological damage. Their ability to preserve cartilage integrity and reduce synovitis was linked to the inhibition of the MAPK/AP−1−MMP9 cascade at the tissue level, as shown by immunohistochemistry. Systemically, the derivatives effectively lowered pro−inflammatory cytokine levels (TNF−α, IL−6, IL−1β), with distinct profiles suggesting structure−dependent modulation of the inflammatory network. Importantly, all derivatives showed a favorable safety profile, with no significant cytotoxicity *in vitro* and minimal hepatorenal toxicity *in vivo*, presenting a clear advantage over methotrexate.

It is important to acknowledge certain limitations of this study. First, pharmacokinetic and hydrolysis data are not included, leaving the *in vivo* conversion efficiency and metabolic fate of these prodrugs to be fully elucidated. Second, while ERK and p38 were investigated, JNK-another MAPK member implicated in MMP regulation-was not examined, suggesting an avenue for more comprehensive pathway analysis in the future. Third, the current safety assessment focused on acute or subacute indicators; long-term toxicological evaluation and deeper investigation into specific immunomodulatory functions (e.g., effects on Th17/Treg balance) would provide a more comprehensive safety and mechanistic profile for clinical translation. These limitations, however, do not undermine the central conclusions but highlight specific areas for further investigation. Future studies should focus on detailed PK/PD profiling, extended mechanistic analysis including JNK signaling, and evaluation in additional RA−relevant cell types to fully characterize the therapeutic potential of these promising derivatives.

In conclusion, the curcumin−borneol ester derivatives developed herein represent a novel and effective strategy for RA treatment. By synergizing the bioactivity of curcumin with the bioavailability−enhancing property of borneol, these compounds achieve potent multi−target inhibition of the inflammatory cascade responsible for joint destruction. This work provides a compelling proof of concept for a natural product−based prodrug approach and warrants further development as next−generation anti−arthritic agents.

## Data Availability

The original contributions presented in the study are included in the article/supplementary material. Further inquiries can be directed to the corresponding author.
